# Efficacy of threading lasso fixation in repairing partial articular supraspinatus tendon avulsion lesions: a retrospective study

**DOI:** 10.1186/s12891-021-04739-y

**Published:** 2021-10-05

**Authors:** Sun-Yu Chen, Zhan-Hao Xiao, Jian-Kun Wang

**Affiliations:** Department of Sports Injury, Fuzhou Second Hospital Affiliated to Xiamen University, No. 47, Shangteng Road, Cangshan District, Fuzhou, Fujian Province 350007 P.R. China

**Keywords:** Arthroscopy, PASTA lesion, Repair, Supraspinatus

## Abstract

**Background:**

The partial articular supraspinatus tendon avulsion (PASTA) lesion repair remains a topic of debate. We have performed in situ repair of PASTA lesions using a potentially viable threading lasso fixation technique. This retrospective case series aimed to evaluate the clinical outcomes of PASTA lesion repair using threading lasso fixation. To the best of our knowledge, this is the first study to review this technique and its outcomes in terms of pain and upper extremity function.

**Methods:**

Twenty-five patients with PASTA lesions who were treated with threading lasso fixation were reviewed. All patients were followed up for at least 1 year. Preoperative and follow-up data were retrospectively collected and reviewed. Clinical outcomes were assessed to evaluate the efficacy of the surgery.

**Results:**

There were no postoperative complications. The average follow-up period was 25.7 (22–27) months. At the last follow-up, all patients underwent follow-up magnetic resonance imaging; only two cases showed a partially healed tendon and no case converted to full-thickness tear. Furthermore, shoulder pain decreased and mobility was recovered, with statistically significant differences in all scoring measures. Specifically, the mean visual analog scale score decreased from 5.4 ± 1.2 before surgery to 1.1 ± 0.8 at the last follow-up (*t *= 14.908, *P* < 0.01), and the mean American Shoulder and Elbow Surgeons Shoulder Assessment Form score improved significantly from 51.6 ± 6.4 to 89.3 ± 5.2 (*t* = 22.859, *P* < 0.01). Additionally, the mean University of California Los Angeles score improved from 17.8 ± 3.5 preoperatively to 32.3 ± 1.4 (*t* = 19.233, *P* < 0.01).

**Conclusions:**

Arthroscopic repair using threading lasso fixation is a novel transtendinous technique for patients with partial articular supraspinatus tendon avulsion. Tendon integrity is preserved with this method, which may result in improved function. Overall, threading lasso fixation technique is an effective treatment.

## Background

Rotator cuff injury is a common clinical diagnosis. Till date, most treatments and research have focused on full-thickness tears of the rotator cuff. A partial-thickness tear is often overlooked, and there are controversies over its appropriate treatment. For example, the necessity and/or timing of repair have not been clarified [1]. Partial-thickness rotator cuff tears are classified into articular-sided, bursal-sided, and intra-tendinous tears. Articular-sided tears are the most common, with an incidence that is approximately 2–3 times higher than that of bursal-sided tears [2, 3]. Patients with partial-thickness rotator cuff tears account for 13–32% of the general population; as age increases, the proportion gradually increases [4].

Not all patients with partial-thickness rotator cuff tears require surgical treatment; however, with the passage of time, 11.7% will eventually require surgical repair [4]. In the past, articular-sided rotator cuff tears were repaired after conversion to full-thickness tears. With advancements in arthroscopic techniques, more clinicians currently use a trans-tendon technique to repair articular-sided partial-thickness tears [5–7].

In this study, we investigated 25 patients with PASTA lesions who were treated with threading lasso fixation technique to preserve the integrity of the bursal-sided rotator cuff tissue. To the best of our knowledge, this is the first study to review this technique and its results in terms of pain and upper extremity function.

## Methods

### Study population

This retrospective case series study was approved by the Ethics Committee of FuZhou Second Hospital (FZEY2014058). Written informed consent was obtained from all patients. All methods were carried out in accordance with relevant national guidelines and regulations. Between March 2016 and April 2018, 25 patients (16 men, 9 women) were admitted and treated for PASTA lesions. Their ages ranged from 45 to 62 years, with an average of 53.2 years. The time to treatment ranged from 6 to 12 months, with an average of 8.3 months.

The inclusion criteria were as follows: shoulder pain, weakness, and other clinical manifestations, with no underlying diseases; shoulder magnetic resonance imaging (MRI) confirmed the diagnosis of PASTA lesions; no improvement after 6 months of conservative treatment; and surgery performed by the same surgeon as for the remaining patients.

The exclusion criteria were as follows: osteoarthritis of the shoulder; cases complicated by severe cardiovascular and cerebrovascular diseases or nervous system diseases; tears accompanied by shoulder dislocation; presence of rheumatoid arthritis; infection around the shoulder joint; and refusal to participate in the study or inability to follow the rehabilitation protocol.

### Surgical technique

**Fig. 1:**
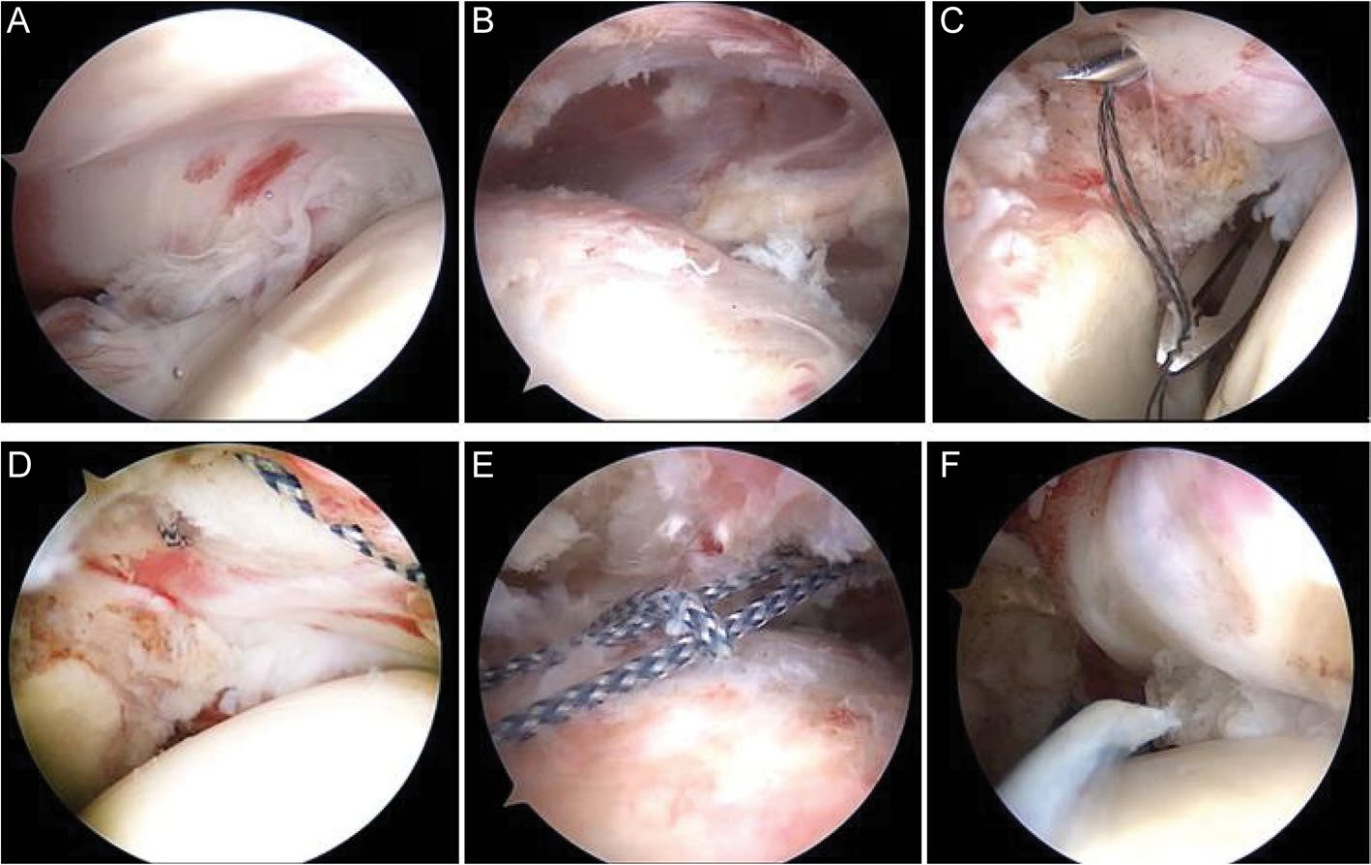
Repair of a PASTA lesion with threading lasso fixation technique. **A** A PASTA lesion. **B** The intact supraspinatus tendon bursa. **C** The thread is passed through the spinal needle. **D** High-strength suture, passing from the articular side of the tendon. **E** High-strength suture, passing from the bursal side to form a lasso. **F** The articular side of the supraspinatus tendon after fixation and repair

**Fig. 2:**
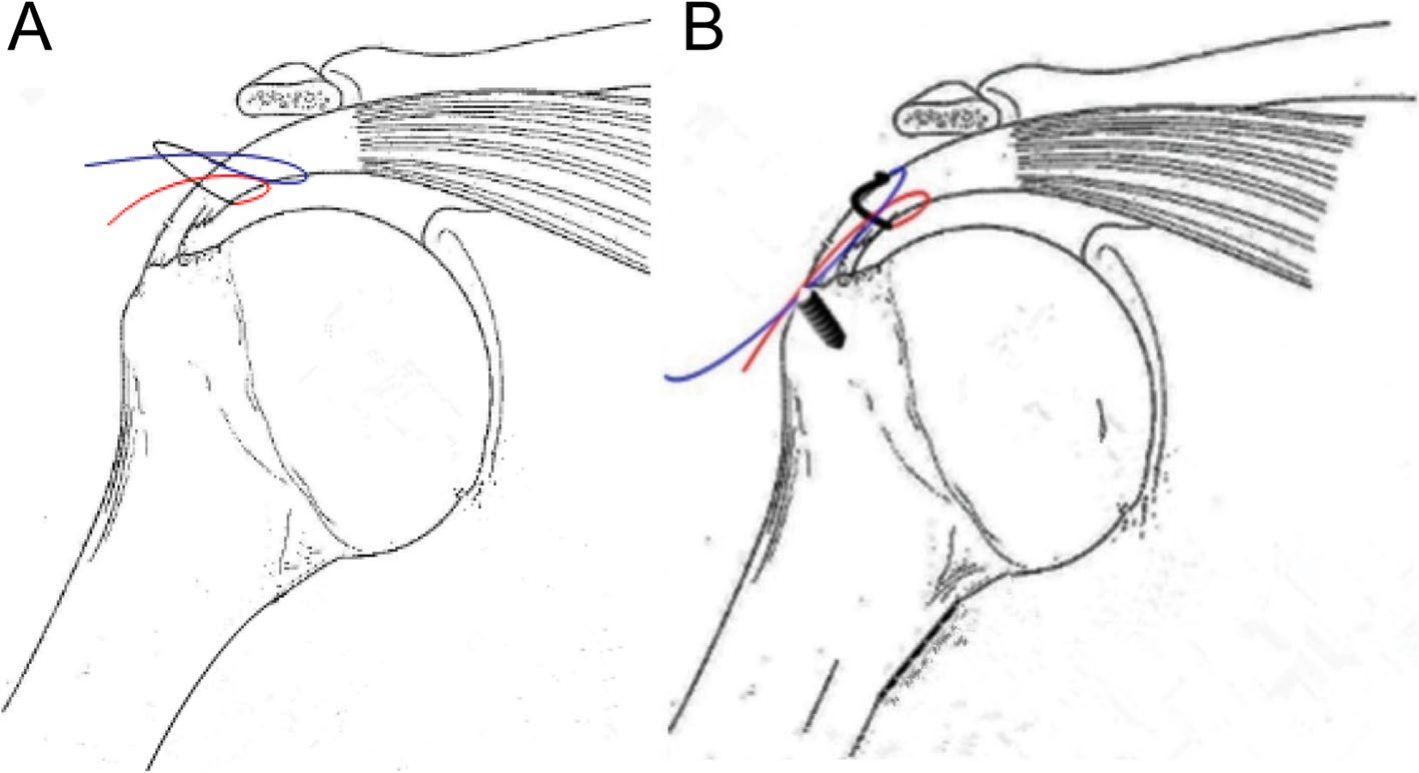
Surgical diagrams. **A** Lasso formation after sutures are passed through the tendon. **B** The sutures are pulled and fixed to the anchors in the greater tuberosity of the humerus

The lateral decubitus position was adopted in all patients, and the surgery was performed under general anesthesia, with endotracheal intubation and brachial plexus block. After anesthesia induction, routine disinfection and draping were performed, followed by traction of the shoulder, with the arm in 45° of abduction and 15° of forward flexion. Anterior, posterior, and anterolateral approaches to the shoulder joint were established, and the glenoid and subacromial spaces were explored to observe the PASTA lesions (Fig. 1A). Damaged tissues were prepared using radiofrequency coblation and a motorized shaver. Debridement of the subacromial bursa was performed after entering the subacromial space. In cases of subacromial impingement, acromioplasty was performed, and the surface of the rotator cuff was further inspected (Fig. 1B). By viewing from the posterior approach and using a spinal needle as a guide to determine the proper position, a lateral approach was established. The spinal needle was passed through both sides of the proximal end of the articular-side injury, along the bursal-sided surface of the supraspinatus tendon, and a thread was passed through the spinal needle (Fig. 1C). A high-strength suture was introduced to form a lasso. Using the same technique, the spinal needle was made to penetrate both sides of the distal end of the articular-side injury, with the suture tails protruding from the bursal side of the supraspinatus tendon and drawn through the lasso using a threader (Fig. 1D and E). After pulling the sutures, a lateral row of anchors was used to fix the sutures to the greater tuberosity of the humerus. We again probed the glenohumeral joint and confirmed that the torn end of the supraspinatus tendon was close to the footprint area (Fig. 1F). Surgical images the revealing areas of importance are provided in Fig. 2A and B.

After the procedure, the involved limb was suspended and fixed with abduction protective gear; passive forward flexion, abduction, and internal and external rotation exercises were started on the first postoperative day (frequency: 5 times per session, 2 sessions per day, with 10 min of icing after the exercises). At 1 month postoperatively, the shoulder abduction protective gear was removed. The range of motion gradually increased to normal, and strength training was initiated.

All patients were assessed using the visual analog scale (VAS), American Shoulder and Elbow Surgeons (ASES) Shoulder Assessment Form, and University of California Los Angeles (UCLA) Shoulder Rating Scale, before the surgery and at the last follow-up after the surgery. To assess tendon healing, anatomical evaluation of the cuff repair was performed using MRI scan as the investigation of choice. Intact cuff with no fluid leakage was considered diagnostic of a “healed tendon”; if the supraspinatus tendon did not heal to the greater tuberosity of the humerus and the size of the persistent defect was smaller than the initial tear, diagnosis of a “partially healed tendon” was made; if the supraspinatus tendon did not heal to the greater tuberosity of the humerus and the persistent defect was converted to a full-thickness tear, diagnosis of a “re-tear” was made [8].

### Statistical analysis

Statistical analyses were performed using Statistical Package for the Social Sciences version 19.0 (IBM Corporation, Armonk, NY, USA). Continuous variables (VAS, ASES, and UCLA scores) were expressed as mean ± standard deviation ($\overline{x}$ ± s). As the sample size was small, normally distributed variables were compared using the paired t-test. The wilcoxon signed rank test was used for non-normally distributed variables. Differences with *P* < 0.05 were considered statistically significant.

## Results

**Fig. 3:**
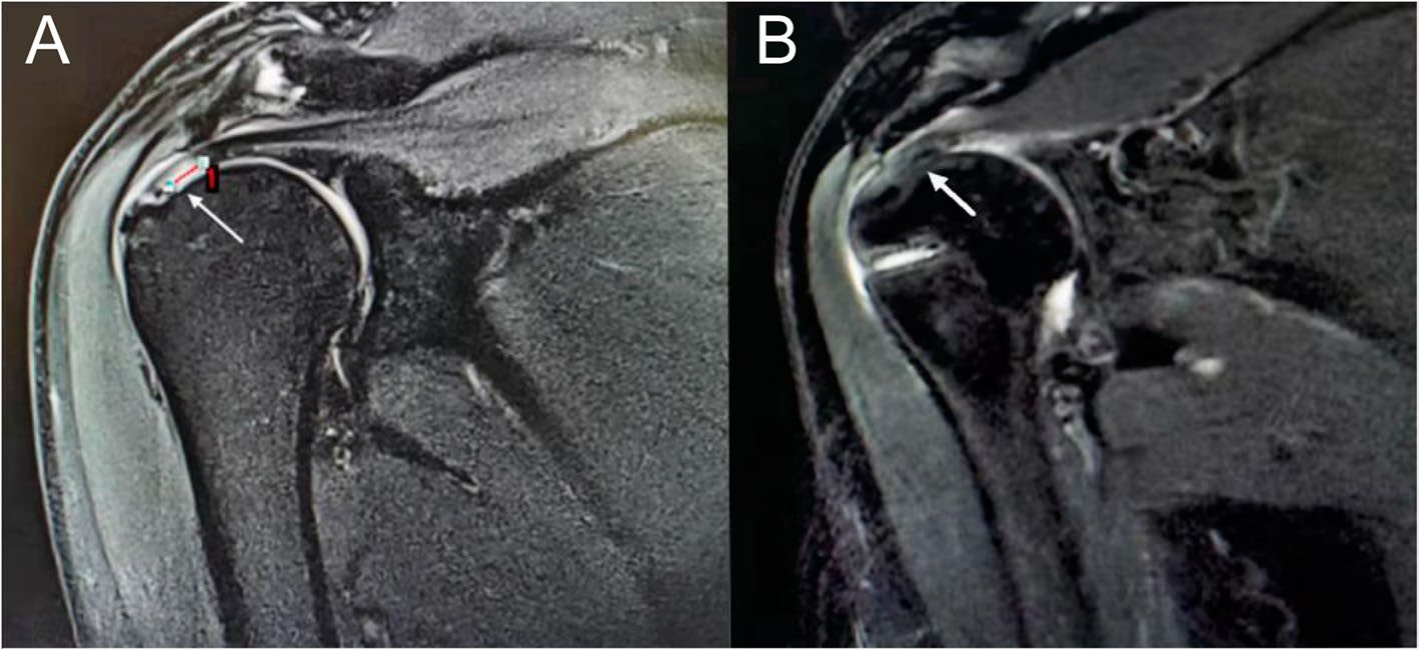
Preoperative and postoperative MRI scans of a patient who had partial articular supraspinatus tendon avulsion. **A** Preoperative MRI of a PASTA lesion. **B** MRI at the last follow-up after repair of a PASTA lesion using the threading lasso fixation technique MRI, magnetic resonance imaging.

The average follow-up period was 25.7 (22–27) months. The average operative time was 1.1 (0.8–1.3) hours. No complications, such as infection, fixture loosening, or joint stiffness, occurred postoperatively. MRI of the shoulder joint was obtained at the last follow-up and compared with the preoperative MRI (Fig. 3). Only two cases showed a partially healed tendon and no case progressed to full-thickness tear. Furthermore, shoulder pain decreased and mobility was recovered; statistically significant differences were observed in all pain and function measures before and after surgery (Table 1). Specifically, the mean VAS score decreased from 5.4 ± 1.2 before surgery to 1.1 ± 0.8 at the last follow-up (*t* = 14.908, *P* < 0.01). The mean ASES score improved significantly from 51.6 ± 6.4 before surgery to 89.3 ± 5.2 at the last follow-up (*t* = 22.859, *P* < 0.01). The mean UCLA score improved from 17.8 ± 3.5 before surgery to 32.3 ± 1.4 at the last follow-up (*t* = 19.233, *P* < 0.01). Two cases showed incomplete tendon–bone healing; however, a good functional score and joint range of motion were still obtained.

**Table 1:** Comparison of preoperative and postoperative clinical data of patients who underwent threading lasso fixation ($\overline{x}$ ± s)

Time	No. of cases	VAS 7score (points)	ASES score (points)	UCLA score (points)
Before surgery	25	5.4 ± 1.2	51.6 ± 6.4	17.8 ± 3.5
At last follow-up	25	1.1 ± 0.8	89.3 ± 5.2	32.3 ± 1.4
*T*		14.908	22.859	19.233
*P*		< 0.001	< 0.001	< 0.001

*VAS* Visual Analog Scale, *ASES* American Shoulder and Elbow Surgeons, *UCLA* University of California Los Angeles

## Discussion

The rotator cuff comprises the subscapularis, supraspinatus, infraspinatus, and teres minor tendons; among these, the supraspinatus tendon is the most vulnerable. The supraspinatus tendon abducts the upper arm following muscle contraction, and the stress is relatively concentrated in the central area of the tendon; thus, a supraspinatus injury is related to the mechanical function and anatomy of the shoulder joint [9]. The supraspinatus tendon can be partially torn due to degeneration, tension, internal impact, and other factors. Over time, conversion to full-thickness tear can occur. The risks of tendon retraction, fat infiltration, and muscle atrophy are relatively low for partial-thickness tears of the supraspinatus tendon, and most partial-thickness tears can be treated non-surgically. Consistent with this, Kong et al. managed 81 patients with partial-thickness tears of the supraspinatus tendon conservatively and found that after 1 year of follow-up, only 13 patients showed tear expansion on MRI [10]. Furthermore, Engelhardt et al. concluded, by means of a finite element analysis, that partial-thickness tears of less than 40% should be treated mainly with rehabilitation, while tears of more than 60% should be surgically repaired to restore shoulder strength [11]. Currently, most clinicians prefer to use surgical intervention in patients with tendon tears of greater than 50% [12–14]. Regarding the timing of surgical treatment, Kim et al. reported that surgical repair after 6 months of conservative treatment was superior to immediate surgical repair for partial-thickness rotator cuff tears [15].

In considering the surgical repair of partial-thickness rotator cuff tears, the depth of the tendon tear and the patient’s symptoms should be comprehensively evaluated [4, 16]. Vap et al. performed arthroscopic repair in patients with partial-thickness tears of the supraspinatus tendon and found that no patient needed revision rotator cuff repair during a 5-year follow-up period; furthermore, the return to activity rate was high after the repair of isolated partial-thickness rotator cuff tears [17]. Current suturing methods for partial-thickness rotator cuff tears include in situ repair and full-thickness suture. In situ repair preserves the tendon stump by using a trans-tendon technique, thus allowing a stronger stress load-bearing capacity at an early stage, with good clinical efficacy [18, 19]. The full-thickness suture method exposes the bone bed by debridement of the damaged tendon stump, and suturing is performed in a standard manner. This method is convenient and easily mastered by most surgeons [20]. In addition, animal experiments have shown that full-thickness suturing has better outcomes than in situ repair, which may be related to the more thorough debridement that is done in full-thickness suturing [21]. However, Jordan et al. reviewed and summarized the clinical efficacy of these two methods and concluded that the trans-tendon technique resulted in more obvious pain and motion limitations at the early stage of recovery, although both methods eventually achieved satisfactory clinical efficacy [22]. Furthermore, in terms of long-term outcomes, the trans-tendon technique does not have an advantage. However, a biomechanical study found that when articular-sided rotator cuff tears were repaired with two suture anchors using an in situ transtendinous technique, there was significantly less gapping during axial cyclic loading of the tendon than there was in complete tears repaired with a double-row construct using four suture anchors [23].

Conventional transtendinous repair necessitates making a hole through the tendon in order to pass an awl, a tap, and an anchor. This causes a certain degree of damage to the bursal side of the supraspinatus tendon. Because of the biomechanical advantages of in situ repair and reduced damage caused by trans-tendon repair under arthroscopy, the present study used a modified in situ repair technique to repair articular-sided tears that retained the bursal side of the supraspinatus tendon. The technique involved threading by using a spinal needle to form a lasso, pulling the articular-sided tear to the insertion point on the humerus, and fixing the tear with a lateral row of anchors. Unlike conventional transtendinous repair, our modified technique does not require a medial row of anchors, thus sparing the bursal side of the supraspinatus tendon from injury. In our modified technique, the tension of the tendon is maintained by traction of the lasso suture to prevent further expansion of the tendon tear. At the same time, the injured portion is placed in close contact with the humeral head by downward pressure on the tendon. The clinical results showed partial tendon-bone healing in two patients. However, based on the clinical follow-up findings, early functional rehabilitation appeared feasible, and satisfactory clinical efficacy was achieved, which may be related to tendon traction and fixation.

In summary, PASTA lesion is a common clinical diagnosis. Conservative treatment should be adopted for asymptomatic patients and patients with mild symptoms. If conservative treatment fails, the tendon tear expands, symptoms worsen, or the quality of life is affected, accordingly, surgical treatment may be considered. Arthroscopic threading lasso fixation is a modified in situ suture technique that preserves the integrity of any intact portion of the supraspinatus tendon and provides a stronger construct than that obtained with conversion to a complete tear. The in situ transtendinous technique allows early functional rehabilitation, with satisfactory clinical outcomes.

The limitations of this study are as follows: (1) the study was retrospective in nature, (2) no relevant research on biomechanics was conducted, (3) the follow-up period was too short to assess the possibility of re-tears, and (4) the sample size was small. Further studies with larger sample sizes, longer follow-up periods, and biomechanical analysis are recommended.

## Conclusion

PASTA lesions affect patients more frequently than other types of rotator cuff tears. There is no consensus in the literature regarding the appropriate surgical technique between full-thickness suturing and a variety of transtendinous repair techniques. Our results indicated good tendon integrity preservation and short-term clinical outcomes with the threading lasso fixation technique. Further exploration of this transtendinous repair technique, using a larger sample size and longer follow-up, is suggested.

## Data Availability

The data that support the findings of this study are available on request from the corresponding author. The data are not publicly available due to patients’ privacy concerns.

## Abbreviations

VAS: Visual analog scale
ASES: American Shoulder and Elbow Surgeons
UCLA: University of California Los Angeles
MRI: Magnetic resonance imaging
PASTA: Partial articular supraspinatus tendon avulsion
